# Hereditary Neuropathy with Liability to Pressure Palsy Presenting as Bilateral Foot Drop

**DOI:** 10.5152/eurasianjmed.2023.21154

**Published:** 2023-02-01

**Authors:** İsmail Koç, Güray Koç, Betül Özenç, Zeki Odabaşı

**Affiliations:** 1 Department of Neurology, Gülhane Training and Research Hospital, Ankara, Turkey; 2 Department of Neurology, Bilkent City Hospital, Ankara, Turkey; 3 Department of Neurology, University of Health Science Gülhane Medical School, Ankara, Turkey

Dear Editor,

Hereditary neuropathy with liability to pressure palsy (HNPP) is a rare, autosomal dominant disorder that typically presents with episodic, multifocal neuropathy, and recurrent transient pressure palsies.^1^ We report a patient who was misdiagnosed as transverse myelitis suggestive of multiple sclerosis based on acute onset bilateral foot drop. However, detailed history, careful examination, and electroneuromyography (ENMG) were consistent with HNPP.

A 22-year-old male developed a sudden onset of weakness in his legs. Imaging of the brain and spine, serum, and cerebrospinal fluid analysis were all normal. Normoactive deep tendon reflexes with acute onset were interpreted as transverse myelitis. He was given methylprednisolone, which had no obvious effect. He was then referred to our clinic and we learned that the patient had cross-legged sitting while playing a computer game for hours. He had bilateral ankle and toe dorsiflexion weakness; however, inversion was normal bilaterally. He had hypoesthesia on the dorsum of both legs. There was no pathological reflex.

Electroneuromyography showed that sensory nerve conduction (SNC) velocities were slowed in the right median and ulnar nerves but not sural nerve. There was a conduction block in both peroneal nerves at fibular head. Motor conduction velocities were slowed at the elbow segment in both ulnar nerves showing cubital tunnel syndrome (Figure 1). Distal motor latencies were prolonged in both median nerves showing carpal tunnel syndrome. F-wave latencies were prolonged in both tibial nerves (Table 1). Electroneuromyography revealed sensorimotor polyneuropathy with multiple compression neuropathies consistent with HNPP, but also sural sparing was observed. Genetic testing was performed and peripheral myelin protein 22 (PMP22) gene analysis showed the typical deletion, 1.5 Mb in the distal region of the locus 17p.11.2. He was diagnosed with HNPP, and he recovered completely after physical therapy.

Hereditary neuropathy with liability to pressure palsy is rarely seen and characterized by recurrent painless peripheral mononeuropathies at entrapment sites secondary to minor trauma or compression. The peroneal nerve is the most common entrapped, nevertheless, multiple compression neuropathy can be seen simultaneously in HNPP. Electrophysiologically, a nonuniform, demyelinating, polyneuropathy pattern with a pronounced distal slowing in some nerves at entrapment sites can be seen.^1^ Bilateral peroneal nerve palsy is seen rarely. Prolonged squatting such as Strawberry picker’s palsy, holding and pressing the lateral aspect of the flexed knees in a supine position such as obstetric or orthopedic surgical process, use of the pneumatic compression devices or casts, excessive and rapid weight loss can be an etiological factor for bilateral peroneal nerve palsy.^2^ Hereditary neuropathy with liability to pressure palsy can mimic some inflammatory neuropathies; a study that included 73 patients with HNPP showed 2 patients were considered as Guillain–Barré Syndrome (GBS) initially. The first patient complained of acute distal paresthesia and paresia in the lower extremities. The second patient, misdiagnosed as GBS, complained of subacute onset quadriparesis for 10 days, and he was given intravenous immunoglobulin (IVIg). However, the patients had not undergone lumbar puncture (LP).^3^ A feature of SNC abnormalities in GBS is reduced amplitude higher in the median, ulnar, or radial nerve compared to the sural nerve, also called sural sparing. We observed sural sparing in ENMG, but LP was not consistent with GBS in our case. Acute and bilateral foot drop can be rarely seen in degenerative spinal diseases such as a posterolateral disc protrusion at the L3-4-5 levels, which can be treated surgically and should be in the differential diagnosis.^4^

To the best of our knowledge, only 2 case reports of bilateral foot drop associated with HNPP were presented in the literature^2,5^ and bilateral foot drop was seen simultaneously in 1 of them.^2^ Misdiagnosis of HNPP may lead to inappropriate treatment with methylprednisolone, IVIg, or plasma exchange and unnecessary procedure like LP.

The patients with HNPP mostly have a heterozygous 1.5-Mb deletion in chromosome 17p11.2, the region that encompasses the gene for PMP22. The episodes of pressure palsies are rarely permanent and the main management of HNPP is the prevention of compression. Tendon transfer surgery or decompressive surgery may be helpful in persistent symptoms like foot-drop or wrist-drop, carpal or cubital tunnel syndrome.^6^

Consequently, in especially younger patients, HNPP should be considered in the differential diagnosis when entrapment neuropathy findings were on more than 1 site in ENMG studies, or recurrent entrapment neuropathies such as foot-drop or wrist-drop, carpal or cubital tunnel syndrome were seen. Early diagnosis and the avoidance of precipitating factors like trauma, skinny clothes, rapid weight loss, prolonged crossed-leg sitting, or leaning on elbows could prevent further progress of the diseaese.^6^

## Figures and Tables

**Figure 1.**
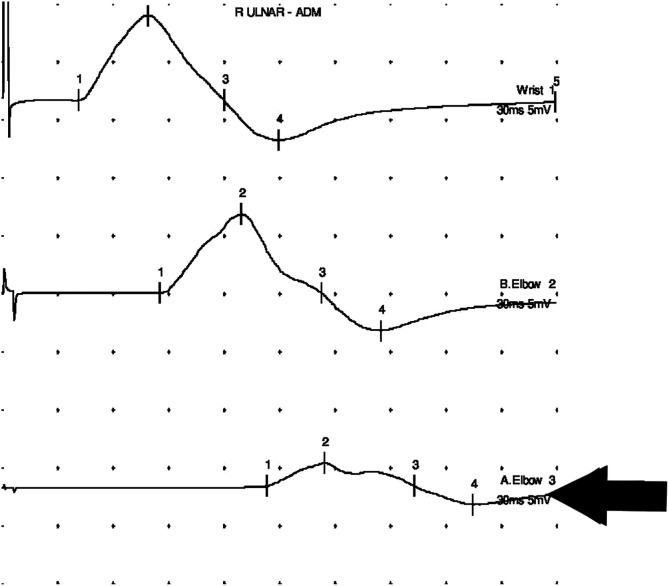
Conduction blocks were detected between the proximal and distal parts of the elbow in the right ulnar nerve (arrow).

**Table 1:** Electroneuromyography

**Sensory NCS**					
**Nerve/Sites**	**Rec. Site**	**Latency (ms)**	**Peak-Base Amplitude (μV)**	**Distance (cm)**	**Velocity (m/s)**
R median – digit II					
1. Wrist	II	**4.15**	**10.0** (>20)	12	**28.9** (>50)
L median – digit II					
1. Wrist	II	3.65	26.3 (>20)	12	**32.9** (>50)
L ulnar – digit V					
1. Wrist	V	2.85	**7.3** (>15)	10	**35.1** (>50)
R ulnar – digit V					
1. Wrist	V	3.55	**10.4** (>15)	12	**33.8** (>50)
L sural - lateral malleolus					
1. Calf	Lateral malleolus	2.10	22.3 (>8)	10	47.6 (>40)
R sural - lateral malleolus					
1. Calf	Lateral malleolus	2.50	19.0 (>8)	11	44.0 (>40)
**Motor NCS**					
**Nerve/Sites**	**Latency (ms)**	**Peak-Base Amplitude (mV)**	**Distance (cm)**	**Velocity (m/s)**	
R median – APB					
1. Wrist	**4.90** (<4)	6.3 (>5)	5		
2. Elbow	10.05	6.4	24	**46.6** (>50)	
L median – APB					
1. Wrist	**4.60** (<4)	9.4 (>5)	5		
2. Elbow	9.45	8.1	24	**49.5** (>50)	
R ulnar – ADM					
1. Wrist	**4.10** (<3)	7.4 (>6)	5		
2. B. elbow	8.50	6.9	22	50.0 (>50)	
3. A. elbow	14.35	**2.0**	10	**17.1**(>50)	
L ulnar – ADM					
1. Wrist	**3.85** (<3)	10.1(>6)	5		
2. B. elbow	8.00	7.5	20	**48.2** (>50)	
3. A. elbow	11.30	7.1	12	**36.4** (>50)	
R common peroneal – EDB					
1. Ankle	**7.20** (<5)	**2.6** (>3)	6		
2. Fibular head	15.85	**2.2 **	33	**38.2** (>40)	
3. Knee		**NP**			
L common peroneal – EDB					
1. Ankle	5.00 (<5)	4.4 (>3)	5		
2. Fibular head	12.65	5.6	33	43.1 (>40)	
3. Knee	15.75	**0.2**	8	**25.8** (>40)	
L tibial (knee) – AH					
1. Ankle	3.85 (<5.8)	11.4 (>4)	6		
2. Knee	13.70	8.4	40	40.6 (>40)	
R tibial (knee) – AH					
1. Ankle	4.90 (<5.8)	11.4 (>4)	6		
2. Knee	14.55	9.2	40	41.5 (>40)	
**F Wave**					
**Nerve**	**Min F latency (ms)**	**Max F latency** **(ms)**	**Mean F latency (ms)**		
R tibial (knee) – AH	**59.10** (<50)	60.80	59.70		
L tibial (knee) – AH	**58.40** (<50)	60.35	59.22		
R median – APB	32.70 (<33)	33.15	32.90		
R ulnar – ADM	**46.85** (<33)	47.10	46.94		

A, Above; ADM, abductor digiti minimi; AH, abductor hallucis; APB, abductor pollicis brevis; B, Below; comm, common; cm, centimeter; L, left; lat, lateral; m/s, meter per second; μV, microvolt; ms, millisecond; NP, no potential; R, right.

Normal values were in parenthesis. Abnormal values were written in bold.
